# Quantitative B-lymphocyte deficiency and increased TCRγδ T-lymphocytes in acute infectious spondylodiscitis

**DOI:** 10.1038/s41598-018-33318-w

**Published:** 2018-10-11

**Authors:** Anna K. Haugaard, Hanne V. Marquart, Lilian Kolte, Lars Peter Ryder, Michala Kehrer, Maria Krogstrup, Ulrik B. Dragsted, Benny Dahl, Ida E. Gjørup, Åse B. Andersen, Peter Garred, Susanne D. Nielsen

**Affiliations:** 1 Viro-Imunology Research Unit, Department of Infectious Diseases, Rigshospitalet, University Hospital of Copenhagen, Copenhagen, Denmark; 2 Department of Clinical Immunology Section 7631, Rigshospitalet, University Hospital of Copenhagen, Copenhagen, Denmark; 3 Department of Pulmonary and Infectious Diseases, Nordsjællands Hospital, Hillerød, Denmark; 4 Department of Infectious Diseases, Odense University Hospital, Odense, Denmark; 5 Department of Infectious Disease, Herlev University Hospital, Herlev, Denmark; 6 Department of Infectious Disease, Roskilde University Hospital, Roskilde, Denmark; 7 Spine Unit, Department of Orthopaedic Surgery, Rigshospitalet, University Hospital of Copenhagen, Copenhagen, Denmark; 8 Texas Children’s Hospital, Baylor College of Medicine, Houston, Texas USA

## Abstract

Acute infectious spondylodiscitis (AIS) is a serious infection of the spine with rising incidence and a mortality of 3–6%. The role of the immune system in AIS is largely unknown. We performed extensive B and T-lymphocyte phenotyping in patients with AIS at diagnosis and after treatment cessation. In this prospective multicentre study, flow cytometric analysis of T and B-lymphocyte subsets was performed in 35 patients at diagnosis and 3 months after treatment cessation. We additionally analysed levels of immunoglobulins and IgG subclasses, serum level and genetic variants of mannose-binding lectin, and somatic hypermutation. A total of 22 (61%) patients had B-lymphocytes below reference limit at baseline, persisting in 7 (30%) patients at follow-up. We found a lower proportion of CD19 + CD27 + IgD+ marginal zone B-lymphocytes and a higher proportion of γδ+ T-lymphocyte receptors compared with controls at both time points. Immunoglobulin levels were elevated at baseline compared to follow-up, and not associated with absolute B-lymphocyte count. In conclusion, a large proportion of AIS patients presented with profound B-lymphocyte deficiency, only partly reversible at follow-up. Identification of immune dysfunction related to AIS may allow for future targeted therapeutic interventions to restore host immunity.

## Introduction

Acute infectious spondylodiscitis (AIS) is a serious infection of the spine, often requiring long term hospitalization. The reported mortality is between 3 to 6%, and up to half of patients undergo surgical intervention ranging from biopsy to stabilization of the spine^1–5^. The incidence of AIS varies between 4–24 cases/million/year and has been reported to be increasing as a consequence of an aging population and improved diagnostic procedures, primarily magnetic resonance imaging (MRI)^6–9^. A range of risk factors have been identified, including diabetes mellitus, intravenous drug use, advanced age, iatrogen immunosuppression, malignancy, chronic renal failure, rheumatologic diseases and liver cirrhosis^3,6,10^. The role of the immune system in acute infectious spondylodiscitis (AIS) is largely unknown, and, to our knowledge, there are no prior studies of the immune system in patients with AIS or osteomyelitis. We hypothesized, that patients with primary AIS share common features of impaired immune function, and with this prospective multicenter study aimed to investigate major B and T-lymphocyte subpopulations, immunoglobulins, serum level and genetic variants of mannose-binding lectin and somatic hypermutation in patients with AIS at diagnosis and after treatment cessation.

## Results

### Clinical characteristics

Clinical data are presented in Table 1. A total of 35 patients and 30 healthy controls were included between December 2011 and December 2012. Nine patients (26%) were lost to follow-up, eight of these (22%) due to transfer out and one (3%) due to loss of contact. One patient was diagnosed with chronic lymphatic leukaemia and excluded from analysis. We found no difference between patients and controls in age (61 (48–66) versus 54 (51–58) years, *P* = 0.15) or gender (female gender in 26% versus 50%, P = 0.08). Patients were included 18 (14–34) days after initiating treatment and follow-up samples were collected 5 (4–6) months from last dose of antibiotics. The microbial aetiology of infection was confirmed in 74% of cases (Table 1). In total, four patients were treated for infection with mycobacteria, two of those culture positive for *Mycobacterium tuberculosis* (TB), one positive for atypical mycobacteria, one with culture-negative AIS treated according to clinical presentation of TB. Due to the small sample size, separate analysis was not performed for this subgroup. On visual examination, patients with TB did not present as collective outliers in any variable investigated (data not shown). A total of 26 patients (72%) had one or more known risk factor for AIS, including six patients (17%) who had received oral prednisolone treatment a median of 16 (8–22) days from inclusion. All patients tested negative for HIV. Patients were treated with intravenous antibiotics for a median of 6 (3–17) weeks. First-line intravenous therapy consisted of a cephalosporin in 16 (46%), a β-lactam penicillin in 8 (23%), or combination therapy with either of those and metronidazole in 3 (9%), an aminoglycoside in 2 (6%), a quinolone in 1 (2%) or vancomycin in 1 (2%) of patients. Subsequent peroral treatment lasted a median of 8 (4–33) weeks, excluding the four patients treated for infection with TB. First-line peroral therapy consisted of β-lactam penicillin in 18 (51%), a quinolone in 4 (11%), phenoxymethylpenicillin in 3 (9%), linezolid in 1 (2%), rifampicin in 1(2%), clindamycin in combination with a quinolone in 1(2%) or a folic acid inhibitor and sulfonamide in 1(2%) of patients, and was unknown for 2 (6%) of patients. The four patients treated for infection with TB were all treated with a peroral 4-drug regimen consisting of isoniazid, rifampicin, pyrazinamide and ethambutol, with discontinuation of pyrazinamide and ethambutol after 8 weeks and a total duration of antibiotic therapy of 26 weeks. Open spine surgery was performed in 16 (46%) patients, biopsy was collected in 6 (17%) and abscess drainage performed in 2 (6%) of patients, while no invasive procedures was performed in 11 (31%) of patients.

**Table 1:** Clinical characteristics at baseline.

	Baseline n = 35
Female gender, n (%)	9 (26)
Age, median years (IQR)	61 (48–66)
**Days from onset of symptoms to (median (IQR)**	
Appropriate intravenous antimicrobials	22 (6–71)
Diagnosis	26 (14–70)
Inclusion in study	52 (32–111)
Immunosuppressive^a^ treatment <4 weeks prior to admission, n (%)	6 (17)
Days from last dose of prednisolone to inclusion in study, median (IQR)	16 (8–22)
**Aetiology, n (%)**	
Monomicrobial	23 (66)
*Staphylococcus aureus*	9 (40)
*Streptococcus* species	3 (13)
*Escherichia coli*	2(6)
Mycobacteriae^b^	4 (11)
Other^c^	6 (26)
Polymicrobial	3 (9)
*Staphylococcus aureus*	1 (33)
*Streptococcus* species	2 (67)
*Escherichia coli*	1 (33)
Other^c^	2 (67)
No confirmed aetiology	9 (26)
**Site of infection, n** (**%)**	
Cervical	2 (6)
Thoracic	11(31)
Lumbosacral	18 (51)
Multiple foci	4 (11)
**Other foci, n** (**%)**	
Abscess formation	24 (69)
Other^d^	3 (9)
**Comorbidities, n** (**%)**	
Single comorbidity	10 (29)
Multiple comorbidities	12 (34)
Diabetes Mellitus type 2	3 (9)
Bone or joint disease	5 (14)
Alcohol abuse	6 (17)
Previous back surgery	4 (11)
Active IV abuse	2 (6)
Other^e^	25 (71)
**Biochemistry at admission**	
Sedimentation rate, mm/h, median (IQR)	76 (54–99)
Elevated (>15 mm/h), n (%)	26/27 (96)
Haemoglobin, mmol/l, median (IQR)	7.3 (6.5–7.8)
Low (<7.3 for females, <8.3 for males), n (%)	25/34 (74)
C-reactive protein, mg/l, median (IQR)	62 (23–124)
Elevated (>10 mg/l), n (%)	31/33 (94)
White blood cell (WBC) count, *10^9^/l, median (IQR)	8.5 (6.8–10.8)
Elevated (>8.8), n (%)	16/35 (46)
Low (<3.5), n (%)	1/35 (3)
Neutrophils, *10^9^/l, median (IQR)	6.0 (4.4–7.3)
Elevated (>7.4), n (%)	6/35 (17)
Low (<1.8), n (%)	2/35 (6)
Monocytes, *10^9^/l, median (IQR)	0.5 (0.4–0.8)
Elevated (>1.1), n (%)	3/31 (10)
Lymphocytes, 10^9^/l, median (IQR)	1.7 (1.18–2.21)
Low (<0.7), n (%)	4/33 (12)
Thrombocytes, 10^9^/l, median (IQR)	289 (231–387)
Elevated (>390), n (%)	8/33 (24)
Low (<145), n (%)	1/33 (3)

For elevated/low levels of a given parameter, counts equal to zero (0%) are not shown. ^a^All patients on immunosuppressive treatment prior to inclusion were treated with prednisolone. ^b^Three cases with *Mycobacterium tuberculosis*, one case with atypical mycobacteria. ^c^*Haemophilus influenzae, Salmonella enterica, Enterococcus faecalis, Haemophilus aprophilius, streptococcus anginosus, citrobakter freundii, Enterobacter cloaca, Klebsiella pneumoniae*. ^*d*^Meningitis, Sepsis, Septic Arthritis. ^e^Cardiovascular disease, chronic lung condition, chronic liver condition, chronic kidney failure, rheumatic diseases, previous malignancies.

### Isolated B- lymphocyte deficiency

Total lymphocyte count at baseline (1.8 (1.4–2.2) * 10^9^ cells/l) was comparable to lymphocyte count at follow-up (1.8 (1.3–2.4) * 10^9^ cells/l). At baseline 2/35 (6%) had lymphocytes below reference level (0.7 * 10^9^ cells/l); at follow-up this was 2/26 (8%) of patients. None had lymphocyte count above reference level (4.9 * 10^9^cells/l).

**Figure 1:**
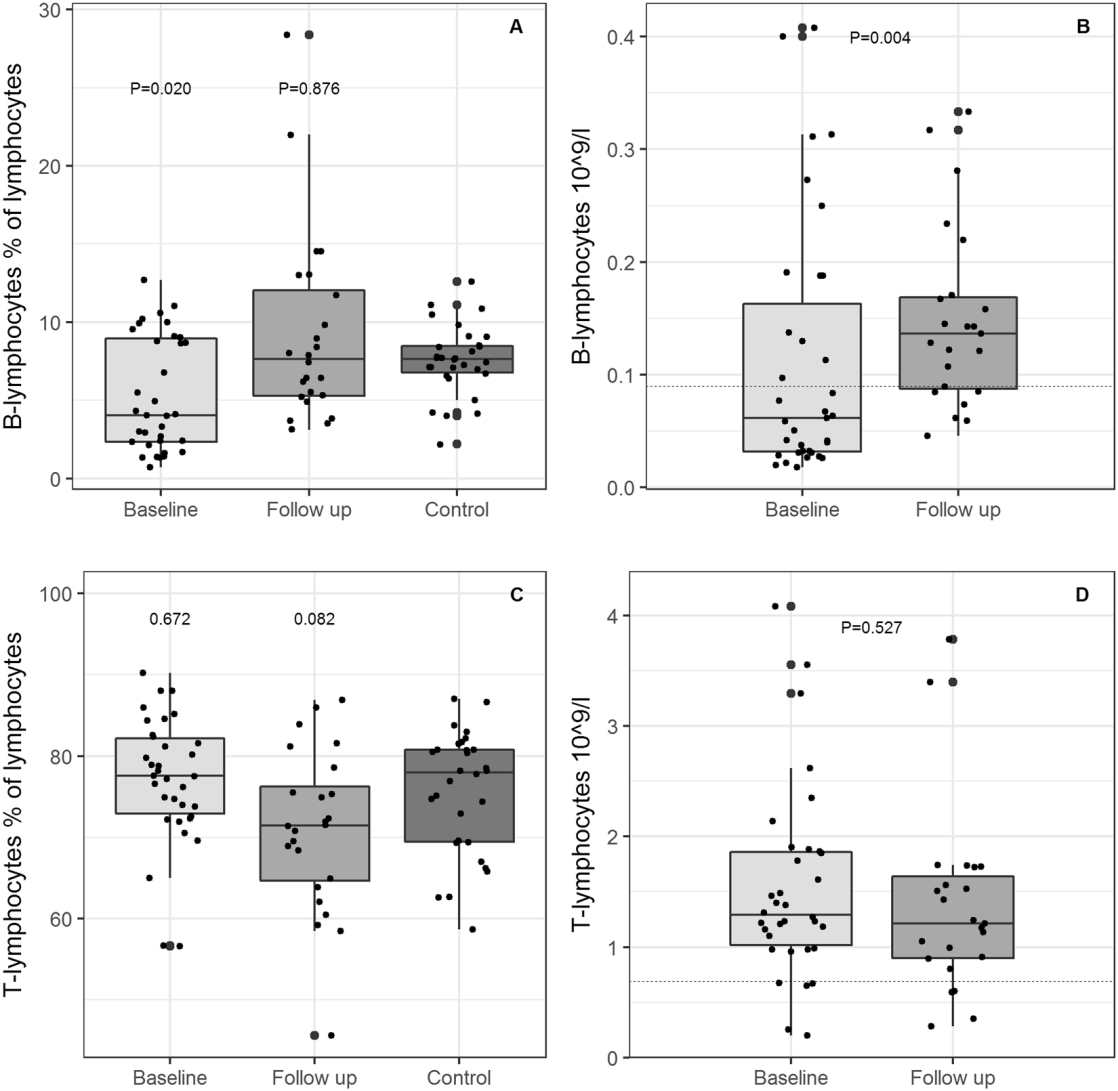
Relative and absolute T and B-lymphocyte counts. (**A**) Proportion of B-lymphocytes at baseline and follow-up, and in controls. (**B**) Absolute B cell count, *10^9^ cells/l. (**C**) Proportion of T lymphocytes at baseline and follow-up, and in controls. (**D**) Absolute T cell count *10^9^ cells/l. P values in (**A** and **C**) are generated from Mann Whitney U test between baseline and controls and between follow-up and controls. P values in (**B** and **D**) are generated from a Wilcoxon signed rank test.

**Figure 2:**
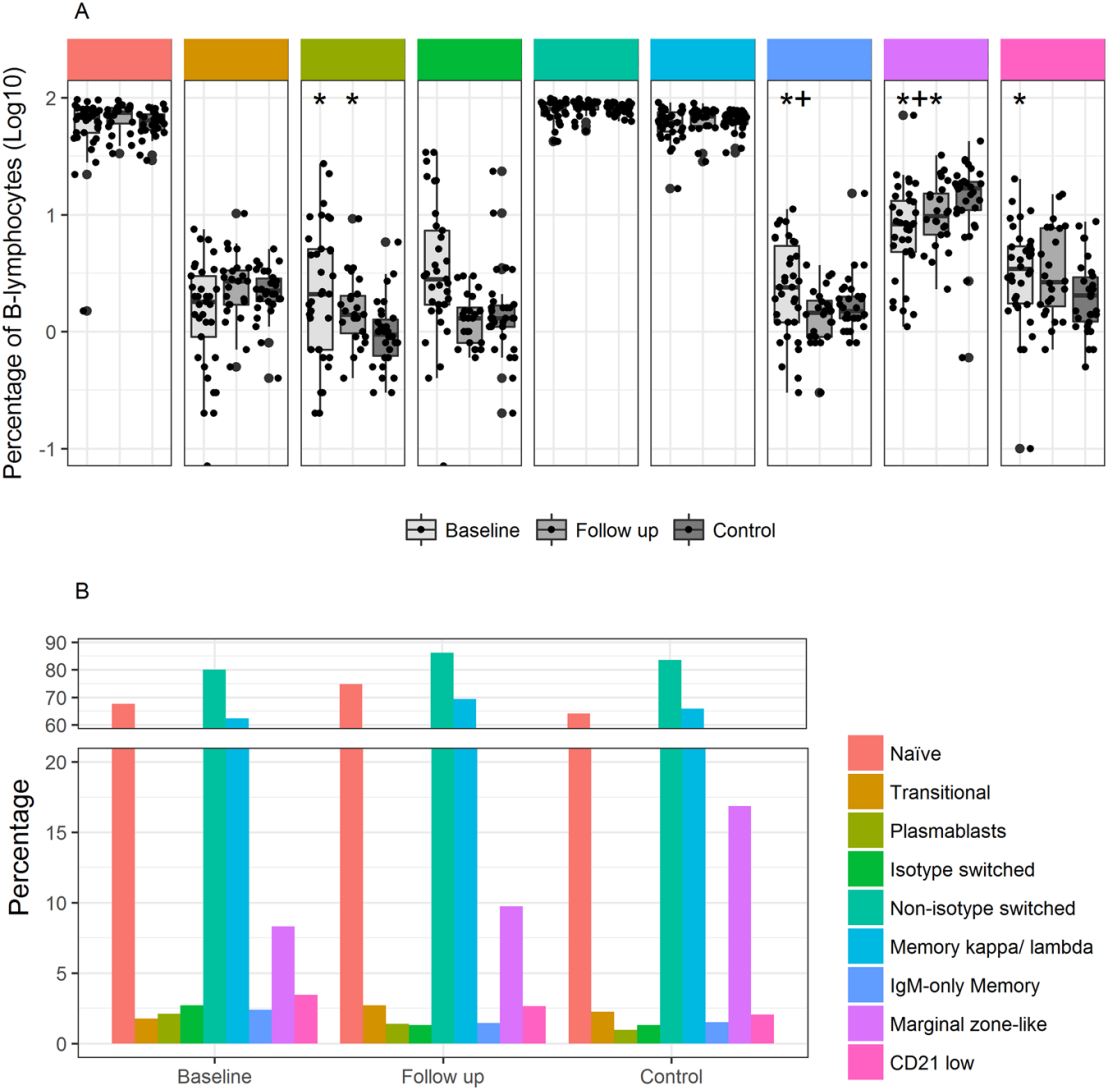
B-lymphocyte subsets (% of B-lymphocytes) in patients at inclusion and follow-up, and in controls. (**A**) Boxplot of B lymphocyte subsets as proportion of B-lymphocytes at baseline, follow-up and in controls. Boxes represent 25^th^ and 75^th^ percentiles, the central line represents the 50^th^ percentile, horizontal lines represent range within lower quartile −1.5 * IQR and upper quartile +1.5 * IQR. Y axis represents proportion of lymphocytes as log10(%). Comparisons were made between patients at baseline and controls, and between follow-up and controls, using the Mann Whitney U test, with significant results demarcated with * and between baseline and follow-up using the paired Wilcoxon signed rank test, with significant results demarcated with+. (**B**) Bar plot showing B-lymphocyte composition in proportion of B-lymphocytes at baseline, follow-up and in controls.

Data from flow cytometric analysis of B-lymphocyte subsets are presented in Table 2 and Figs 1 and 2. An overview of analysed subsets is available as Supplementary Table S1.

**Table 2:** Absolute cell count of B and T-lymphocyte subpopulation and immunoglobulin level in patients at baseline and follow-up, and MBL serum level and genotype at baseline.

	Baseline N = 35	Follow-up N = 26	*P*
**B-lymphocyte subsets**, *10^9^cells/l			
Naïve	0.04 (0.02–0.10)	0.1093 (0.06–0.14)	<***0.01***
Transitional	0.0012 (0.0004–0.003)	0.003 (0.002–0.005)	***0.001***
Plasmablasts	0.002 (0.001–0.004)	0.0019(0.0013–0.0027)	0.57
Memory	0.0300 (0.0193–0.0978)	0.0967 (0.0589–0.1328)	***0.01***
IgM-only memory	0.0017 (0.0009–0.0027)	0.0017 (0.0012–0.0034)	0.32
Marginal zone	0.0069 (0.0017–0.0095)	0.0098 (0.0069–0.0209)	***0.001***
CD21 low	0.0022 (0.0014–0.0038)	0.0039 (0.0027–0.0060)	<***0.001***
**Functional test of B-lymphocytes**			
Somatic Hypermutation, % of	67 (49–73)	—	—
Below reference level of 27%,n (%)	1(3)	—	—
**T-lymphocyte subsets**, cells × 10^9^/l			
CD4	0.74 (0.55–1.09)	0.80 (0.49–1.01)	0.95
CD8	0.40 (0.28–0.67)	0.35 (0.23–0.52)	0.95
TCRαβ	1.14 (0.96–1.70)	1.13 (0.83–1.48)	0.46
TCRγδ	0.07 (0.03–0.15)	0.06(0.04–0.11)	0.61
Memory CD4	0.42 (0.23–0.60)	0.45 (0.26–0.57)	0.87
Memory CD8	0.14 (0.07–0.21)	0.09 (0.06–0.18)	0.95
Recent thymic emigrants	0.09 (0.06–0.16)	0.1127 (0.05–0.16)	0.22
Naïve CD4	0.27 (0.15–0.45)	0.28 (0.15–0.40)	0.85
Naïve CD8	0.06 (0.02–0.09)	0.04 (0.02–0.07)	0.57
Effector memory CD4	0.14 (0.08–0.22)	0.1224 (0.08–0.20)	0.73
Central Memory CD4	0.30 (0.16–0.38)	0.29 (0.17–0.41)	0.85
Effector memory CD8	0.21 (0.13–0.34)	0.18 (0.10–0.30)	0.90
Central Memory CD8	0.06 (0.02–0.09)	0.05 (0.02–0.06)	0.95
Activated CD4 cells	0.03 (0.02–0.04)	0.02 (0.01–0.04)	0.29
Activated, exhausted CD4	0.03 (0.02–0.05)	0.0152 (0.01–0.03)	**0.05**
Activated CD8 cells	0.06 (0.03–0.13)	0.0290 (0.01–0.05)	**<0.05**
Activated, exhausted CD8	0.02 (0.01–0.04)	0.01 (0.00–0.01)	**<0.001**
Tc17	0.02 (0.01–0.02)	0.01 (0.01–0.03)	0.37
Th17	0.03 (0.01–0.05)	0.03 (0.01–0.07)	0.39
Treg	0.06 (0.04–0.08)	0.06 (0.04–0.08)	0.39
**Stimulated proliferation of** CD4+ T**-lymphocytes**			
Proliferation relative to controls, %	99 (96–101)		
Proliferation below reference level, n(%)	1 (3%)		
**MBL**			
MBL, serum level	1971 (59–5670)		
Serum level below 100/below 500, n/n	3/3		
MBL genotype with low MBL, n (%)	7 (20)		
MBL genotype with very low MBL, n (%)	3 (9)		
**Immunoglobulins**			
IgM, g/l	1.2 (0.6–1.9)	0.9 (0.5–1.2)	** < 0.01**
Below reference level, n/total	1/31	1/24	
IgA, g/l	2.8 (2.0–4.2)	2.2 (1.8–3.1)	**0.02**
Below reference level, n/total	0/28	1/24	
IgG total, g/l	12.6 (10.0–16.0)	10.4 (9.3–13.2)	**0.02**
Below reference level, n/total	0/32	0/24	
IgG1, g/l	7.0 (4.8–11.2)	6.2 (5.2–8.0)	0.31
Below reference level, n/total	1/25	0/23	
IgG2, g/l	4.0 (2.7–5.1)	3.2 (2.6–3.9)	**0.01**
Below reference level, n/total	1/25	2/23	
IgG3, g/l	0.8 (0.5–1.0)	0.5 (0.4–0.7)	**0.001**
Below reference level, n/total	2/25	4/23	
IgG4, g/l	0.4 (0.2–0.8)	0.5 (0.3–0.9)	**0.04**
Below reference level, n/total	3/25	3/23	

Comparisons are generated from paired Wilcoxon signed rank test. Abbreviations: TCRαβ, αβ T-lymphocyte receptor; TCRγδ, γδ T-lymphocyte receptor; MBL, mannose binding lectin.

The proportion of B-lymphocytes was lower at baseline than the proportion in controls. At follow-up, this difference was not found (Fig. 1). Accordingly, at follow-up, patients had a significantly higher B-lymphocyte count than at baseline. A total of 22 patients (61%) had B-lymphocytes below reference limit at baseline, and 7 patients (30%) remained below reference limit at follow-up. The proportion of B-lymphocytes was negatively associated with age (Spearman’s ρ = −0.47, P = 0.005), but absolute B- lymphocyte count was not (Spearman’s ρ = −0.27, P = 0.12).

Interestingly, we found a lower proportion of marginal zone B-lymphocytes in patients both at baseline and follow-up, compared to controls (Fig. 2). The proportion of marginal zone B- lymphocytes, however, was significantly higher at follow-up than at baseline, as was the absolute level of marginal B-lymphocytes.

Patients at baseline and follow-up were comparable to controls when comparing proportion of naïve, transitional and memory B-lymphocytes, as was the ratio between isotype shifted and non-isotype shifted B-lymphocytes (Fig. 2). In accordance with this, comparing baseline with follow-up, we found no difference in proportion of naïve, transitional and memory B-lymphocytes. However, for these subpopulations, the absolute cell count was higher at follow-up compared to baseline (Table 2). A higher proportion of B lymphocytes were plasmablasts in patients at baseline than in controls and although there was a trend towards normalization, this difference remained significant at follow-up (Fig. 2). No difference in proportion of plasmablasts or in absolute cell count was found between baseline and follow-up.

We found no difference in proportion of memory B-lymphocytes between patients and controls or in patients between baseline and follow-up (Fig. 2). In absolute cell counts, memory B-lymphocytes were higher at follow-up than at baseline. Patients had a higher proportion of IgM-only memory B-lymphocytes at baseline than at follow-up and in accordance with this the proportion was higher at baseline than in controls. In absolute cell counts, however, there was no difference between IgM-only memory B-lymphocyte counts at follow-up and at baseline. At baseline, patients had higher proportion of CD21low B-lymphocytes than controls but not at follow-up. We found no difference between baseline and follow-up in proportion of CD21low B-lymphocytes, but the absolute count was significantly higher at follow-up. In general, the absolute lymphocyte count was higher at follow-up than at baseline for all B-lymphocyte subsets except for plasmablasts and IgM-only memory B-lymphocytes.

We quantified circulating immunoglobulins, including IgG subtypes, in patients at baseline and at follow-up (Table 2). Concentration of IgA, IgM, total IgG and IgG1, IgG2 and IgG3 subtypes were higher at baseline than at follow-up. A lower concentration of IgG4 was found at baseline than at follow-up. At follow-up, 7 of 24 (29%) patients who were available for follow-up had concentration below reference limit of at least one type or subtype of immunoglobulin. All of these seven patients had normal absolute counts of B-lymphocytes at follow-up (Supplementary Table S2). We found no correlation between relative or absolute B-lymphocyte count and any type or subtype of immunoglobulin at baseline or follow-up, or with somatic hypermutation at baseline (data not shown).

Somatic hypermutation was evaluated in patients at baseline. Only 1 (3%) had a proportion below the lower reference limit applied in our laboratory.

### Higher fraction of γδ T-lymphocyte receptors in patients than controls

**Figure 3:**
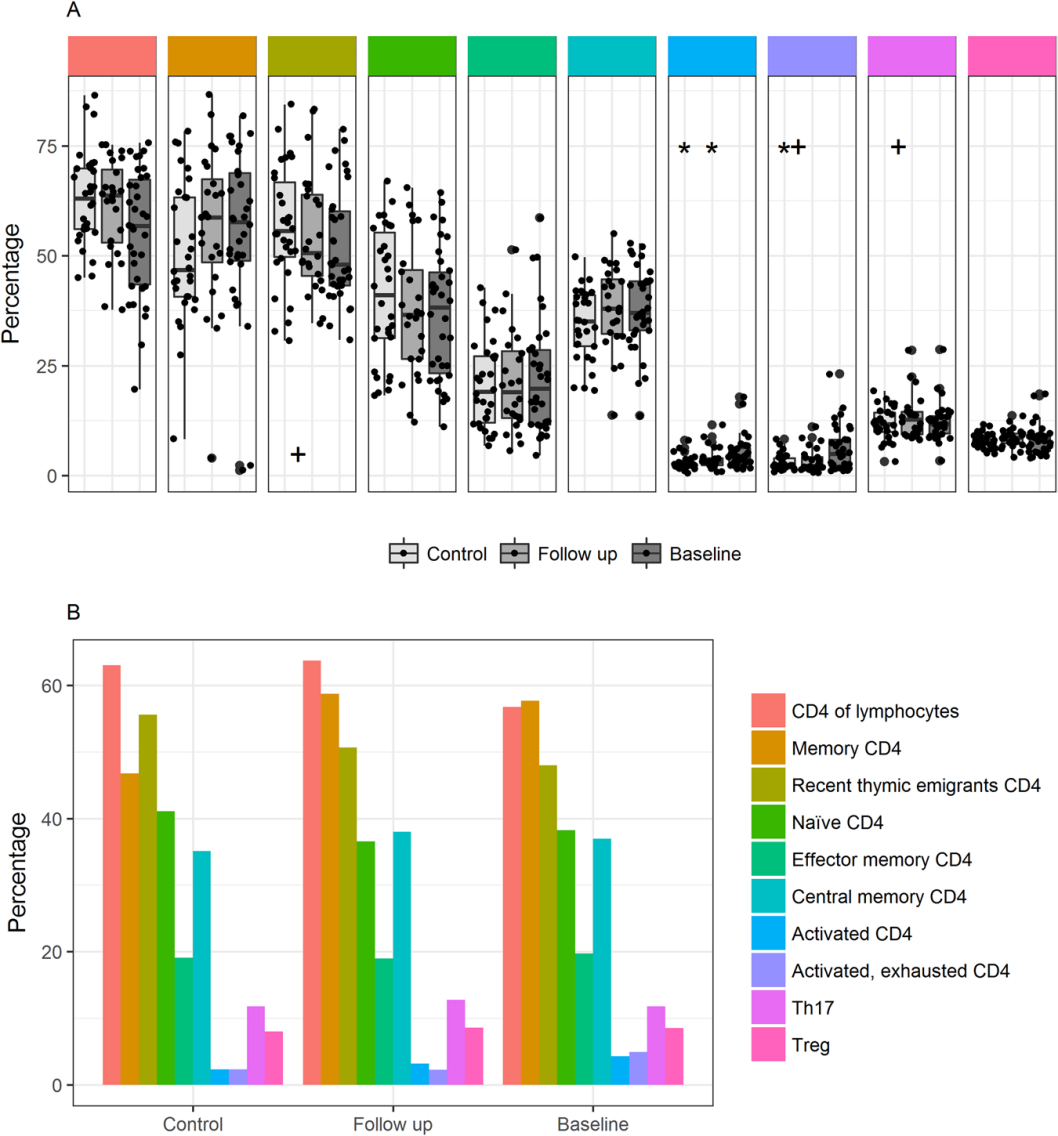
CD4 T-lymphocyte subsets (% of CD4 T-lymphocytes) in patients at inclusion and follow-up, and in controls. (**A**) Boxplot of CD4 T-lymphocyte subsets as proportion of CD4 T-lymphocytes at baseline, follow-up and in controls. Boxes represent 25^th^ and 75^th^ percentiles, the central line represents the 50^th^ percentile, horizontal lines represent range within lower quartile −1.5 * IQR and upper quartile +1.5 * IQR. Y axis represents proportion of lymphocytes. Comparisons were made between patients at baseline and controls, and between follow-up and controls, using the Mann Whitney U test, with significant results demarcated with * and between baseline and follow-up using the paired Wilcoxon signed rank test, with significant results demarcated with+. (**B**) Bar plot showing CD4 T lymphocyte composition in proportion of CD4 T lymphocytes at baseline, follow-up and in controls.

**Figure 4:**
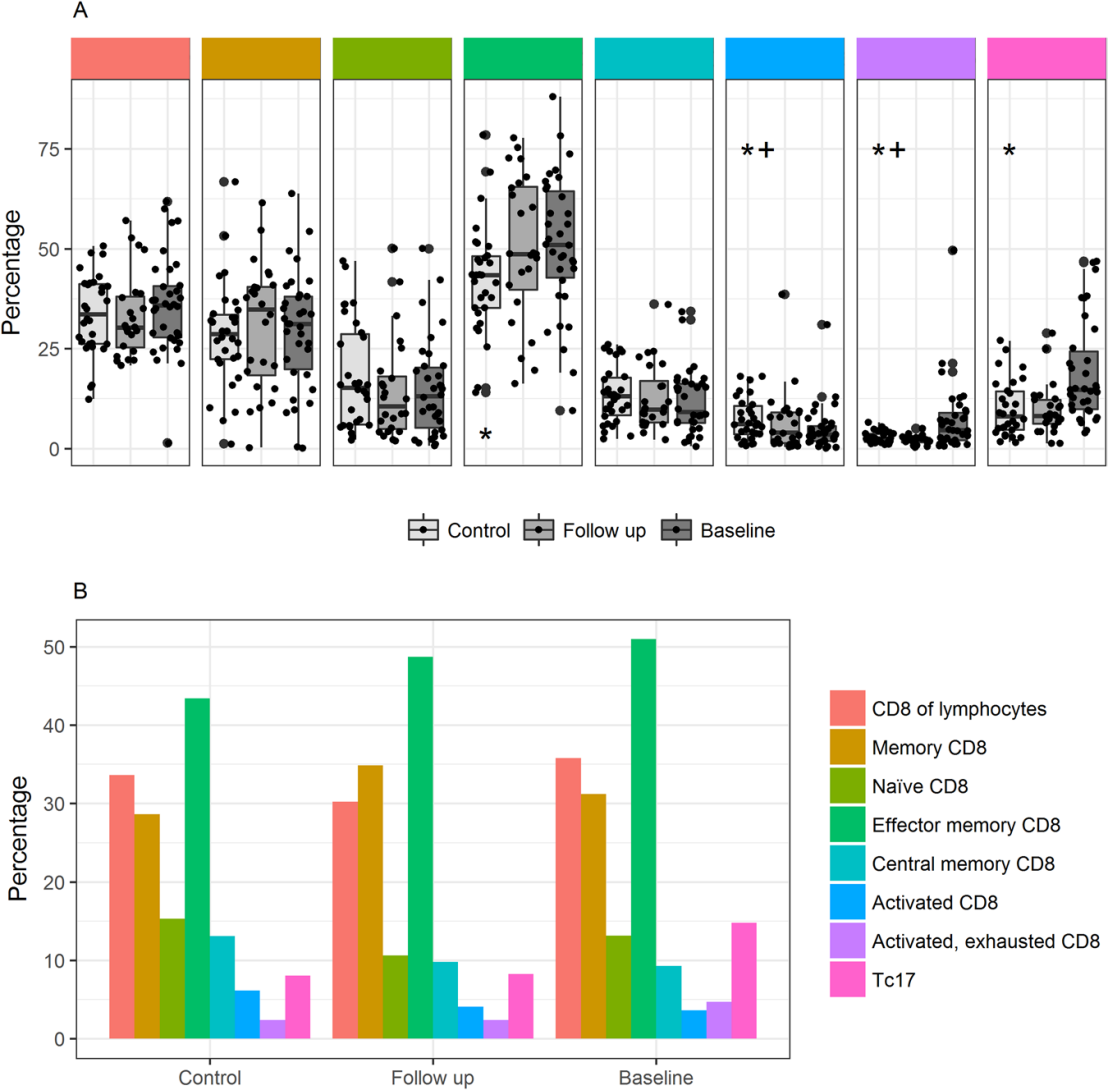
CD8 T-lymphocyte subsets (% of CD8 T-lymphocytes) in patients at inclusion and follow-up, and in controls. (**A**) Boxplot of CD8 T-lymphocyte subsets as proportion of CD8 T-lymphocytes at baseline, follow-up and in controls. Boxes represent 25^th^ and 75^th^ percentiles, the central line represents the 50^th^ percentile, horizontal lines represent range within lower quartile −1.5 * IQR and upper quartile +1.5 * IQR. Y axis represents proportion of lymphocytes. Comparisons were made between patients at baseline and controls, and between follow-up and controls, using the Mann Whitney U test, with significant results demarcated with * and between baseline and follow-up using the paired Wilcoxon signed rank test, with significant results demarcated with+. (**B**) Bar plot showing CD8 T-lymphocyte composition in proportion of CD8 T lymphocytes at baseline, follow-up and in controls.

**Figure 5:**
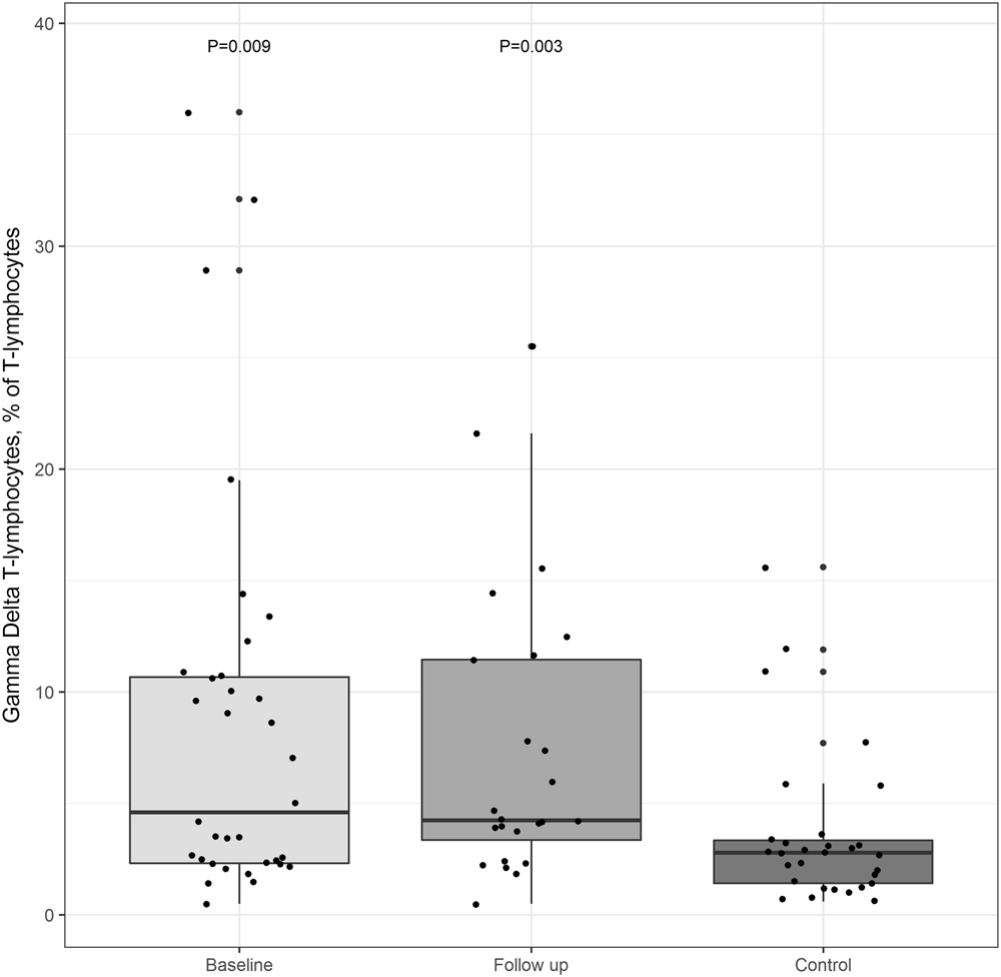
TCRγδ T-lymphocytes. TCRγδ T-lymphocytes at baseline and follow-up and in controls (% of lymphocytes). P values derived from Mann Whitney U-test represent comparisons between patients at baseline and controls and between patients at follow-up and controls.

Data from flow cytometry analysis of T-lymphocyte subsets are presented in Table 2 and Figs 3–5. There was no difference in the proportion of T-lymphocytes between patients and controls at baseline (67.6% (63.6–72.6) versus 67.3% (62.2–74.1), P = 0.67) or at follow-up (64.0% (57.1–68.5), P = 0.08). We found no difference in the absolute T-lymphocyte count in patients between baseline and follow-up (1.2 (0.91.6) * 10^9^cells/l versus 1.1 (0.9–1.4) * 10^9^cells/l).

Patients had a higher proportion of γδ T-lymphocyte receptors (TCRγδ) relative to αβ T-lymphocyte receptors than controls, and this was consistent at follow-up (*P* = 0.006 and *P* = 0.003) (Fig. 5). We found no difference between baseline and follow-up in proportion or absolute level of TCRγδ T-lymphocytes.

### Activated, exhausted T-lymphocyte compartment at baseline

At baseline, patients had a higher proportion of effector memory CD8 T-lymphocytes, a higher proportion of activated CD4 and CD8 T-lymphocytes and a higher proportion of activated exhausted CD4 and CD8 T-lymphocytes than controls (Figs 3 and 4). For these populations, differences from controls were reversible at follow-up, with the exception of activated CD4 T-lymphocytes that remained significantly higher in patients than controls at follow-up. In accordance with this, the proportion and absolute levels of activated CD8 and activated exhausted CD4 and CD8 T-lymphocytes was higher at baseline than at follow-up. Patients had a lower proportion of CD8 Tc17 T-lymphocytes than controls, and this difference was not found at follow-up. At baseline, the proportion of both recent thymic emigrants and Th17 T-lymphocytes was lower than at follow-up (Fig. 3), however, we found no difference between baseline and follow-up in the absolute cell count (Table 2).

In proportions of CD4, CD8, memory CD4, memory CD4/CD8, recent thymic emigrants, naïve CD4/8, CD4 effector memory, CD4/CD8 central memory and Th17 T-lymphocytes we found no differences between patients and controls at baseline or follow-up (Figs 3 and 4). *In vitro* CD4 T-lymphocyte proliferation after CD3/CD2/CD28 cross-linking was evaluated in patients at baseline. Only 1 patient (3%) had a proportion below the lower reference limit applied in our laboratory, and for this patient, results bordered on normal (Table 2).

## Discussion

In this prospective study, we evaluate B and T-lymphocyte subsets in patients with AIS at diagnosis and 3 months after antibacterial treatment. To our knowledge, there are no prior studies of the immune system in patients with AIS or osteomyelitis, although there are casuistic reports of rare cases leading to evaluation of immunologic status^11–13^ and in chronic osteomyelitis, studies have dealt with the interaction between immunological cells, bone resorption and treatment effect^14–18^.

We demonstrated a significant deficiency of B-lymphocytes in patients with AIS. This B-lymphocyte deficiency was only partly reversible, and 3 months after cessation of antibiotic treatment 27% of patients had absolute B-lymphocyte count below threshold. Absolute total lymphocyte and T-lymphocyte counts were comparable to controls. However, the T-lymphocyte compartment in patients had a higher degree of TCRγδ T-lymphocytes than in controls. Not surprisingly, at baseline we found evidence of immune activation and exhaustion in both B and T-lymphocyte compartments.

B-lymphocyte depletion could be caused by lack of production, increase of turnover, compartmentalization or a combination. Lymphopenia in relation to prolonged infection is well described. Immunosuppression is a hallmark of sepsis, and depletion of both B and T-lymphocytes has been reported to be associated with duration of infection and predict early and late mortality^19–21^. One study examined the spleens of septic patients and reported a profound loss of both B and CD4+ T-lymphocytes associated with general lymphopenia^22^. However, although sepsis is an excellent model for generalized inflammation, there are significant differences complicating the direct translation to localized infection. Among these, ischemia/reperfusion injury in sepsis, a possible cause of increased apoptosis, is not commonly reported in AIS^22^. This is supported by our findings of normal absolute levels of lymphocytes and CD4+ and CD8+ T-lymphocytes, isolating the deficiency to the B-lymphocyte compartment. B-lymphocyte dynamics in bacterial, fungal and viral infection have recently been revised, finding similarities in immunologic dysfunction in infection with *Plasmodium falciparum* or *vivax*, *Schistosomia haematobium*, TB, HIV-1 and Hepatitis B and C virus^23^. B-lymphocyte subsets, including marginal zone and naïve B-lymphocytes, have been shown to decrease as a response to these clinically very distinct infections. We found lower levels of marginal zone B-lymphocytes both at baseline and follow-up. Marginal zone dysfunction has also been linked to age related immune dysfunction predisposing to bacterial infection^23^. While the relative frequency of marginal zone B-lymphocytes is too small to account for the general B-lymphocyte deficit, it could reflect a general dysfunction in this population of patients related to age.

Decline in adaptive immune function in elderly patients is well documented^24–26^. Age related immunologic changes include loss of recent thymic emigrants and T-lymphocyte repertoire, poor T-lymphocyte helper function for B-lymphocytes and gradual decrease of the B-lymphocyte pool accompanied by overall increase of low-affinity antibodies and decrease in humoral response to infection^25,27^. We found a negative association between age and relative B-lymphocyte count, indicating that age would seem to impact the B-lymphocyte depletion in these patients. Further studies would be needed to clarify if these results reflect a dysfunctional immunologic response to invasive, prolonged infection in an aging, comorbid population.

We found elevated levels of immunoglobulins at baseline compared to follow-up, and normal somatic hypermutation in circulating B-lymphocytes. However, we did not find the absolute number of plasmablasts to be elevated in baseline compared to follow-up and the number of B-lymphocytes in general was low. B-lymphocytes are thought to compartmentalize in the peripheral lymphoid organs, and plasmablasts migrate to the bone marrow during infection, releasing antibodies into the circulation^28^. It is possible, that the discrepancy between immunoglobulins and plasmablasts at least in part reflects compartmentalization of the B-lymphocyte subsets.

We found a higher proportion of activated CD4+ T-lymphocytes relative to controls at both baseline and follow-up. Interestingly, we found no difference between baseline and follow-up for neither proportion or absolute number of activated CD4+ T-lymphocytes, as would perhaps be expected. Proliferation in response to stimulation of CD4+ T-lymphocytes was normal in all but one patient at baseline. These results could be explained by several factors. It is recognized that the systemic response to an invading pathogen induces both pro- and anti-inflammatory responses, although the timing of the related immunosuppression and its effect on specific subsets remains poorly characterized in humans^29^. Thus, the lack of increased absolute levels of activated CD4+ T-lymphocytes at baseline could be caused by a general anti-inflammatory response. Furthermore, as we found a difference in proportion relative to healthy controls, the lack of difference between baseline and follow-up could in part reflect immune activation that has not subsided significantly 3 months post treatment cessation.

Interestingly, we found patient T-lymphocytes to display a TCRγδ more often than controls, and this was persistent through follow-up. A high proportion of circulating TCRγδ T-lymphocytes could reflect a preexisting condition explaining an inherent vulnerability to bone infection, or the immunologic response to infection of the bone tissue compartment. The TCRγδ T-lymphocytes are a unique subset of lymphocytes normally comprising 1–10% of circulating T-lymphocytes. While their role in host defense against infections remains to be fully elucidated, distinct features have been described, including anatomical compartmentalization in tissues and specific effector functions in both the early and late phase of immune responses. The latter includes regulation of B-lymphocytes and stromal cell function via insulin-like growth factor 1^30^. TCRγδ T-lymphocytes have been found disproportionately responsive to distinct infections, including CMV, TB, Human Immunodeficiency Virus and *Plasmodium falciparum*, and TCRγδ T-lymphocyte deficiency has been shown to increase susceptibility to a range of bacterial infections^31,32^. Interestingly, one recent study found that TCRγδ T-lymphocytes were the major source of IL-17A in bone injury, promoting osteoblastogenesis and bone regeneration^32^. It is possible, that the high proportion of TCRγδ T-lymphocytes in patients with AIS reflects a distinct role for these cells in infection of the bone that persists long into the phase of tissue regeneration and healing.

## Limitations

There are several important limitations to this study. Our study design limits conclusions on cause and effect, as ideally, evaluation of the immune system should precede diagnosis of AIS. *S. aureus* was identified in 9 (40%) of all cases, while this number is generally reported to be around 60%^1^. We included patients consecutively from one tertiary and 4 secondary admission hospitals. It is possible that this reflects a bias in our cohort. Furthermore, follow-up was performed a minimum of three months after cessation of antibiotic treatment to allow for immune reconstitution. However, data on kinetics in immune cell subsets after prolonged (bone) infection is not available and three months might be too early to account for full immune reconstitution^33^. Thus, the results of this study could well reflect prolonged effects of infection on the immune system, rather than a preexisting condition prior to AIS^34^. Patients and controls likely differed in degree of comorbidities, life style and nutritional status, complicating interpretation of the results^35^. Thus, the findings of this study must be viewed cautiously.

## Conclusion

In conclusion, this study is the first to provide a detailed insight into the status of the immune system during and after AIS. Identification of immune dysfunction related to AIS may allow for future targeted therapeutic interventions to restore host immunity. Further studies are warranted to elucidate links between host immune response, long term immune dysfunction, bone healing, and clinical outcome in AIS.

## Methods

This was a prospective, observational, case-control study. Patients were enrolled consecutively between December 2011 and December 2012 from 5 Danish centers; Rigshospitalet University Hospital, Hvidovre University Hospital and Herlev University Hospital, the Capital Region of Denmark, Roskilde University Hospital, the Region Zealand and University Hospital of Odense, Region of Southern Denmark. The study was performed in accordance with the Declaration of Helsinki and approved by the Danish Data Protection Agency (30-0695) and The Ethical Committees of the Capital Region of Denmark (H-4-2011-162). Oral and written informed consent was obtained prior to inclusion.

Inclusion criteria were primary AIS verified by magnetic resonance imaging (MRI)^36^. Patients with spondylodiscitis secondary to spinal procedures were not included. Whole blood was drawn at inclusion and three months after last treatment with antibiotic. In case of surgical intervention at time of inclusion, blood analysis at baseline was postponed for 2 weeks to avoid immune modulation caused by the surgical procedure. Control material from healthy blood donors was drawn on the day of patient baseline analyses and selected to match for age and gender.

Flow cytometric analyses of T and B-lymphocytes were performed in patients and controls. Quantitation of lymphocytes, immunoglobulins and serum level of mannose binding lectin (MBL) as well as MBL genetic variants and level of somatic hypermutation was analysed only in patients. These are routine analyses in our laboratory with available reference material.

### Eight-colour flow cytometry

Peripheral blood mononuclear cells (PBMC) were isolated from venous blood using density gradient centrifugation by Lymphoprep^TM^ (Axis Sheild, Oslo, Norway), and analysed immediately. PBMCs were stained with antibody combinations identifying T and B-lymphocyte subsets. Overview of flow cytometry panels is available as Supplementary Table S3. Eight-colour flow cytometric immunophenotyping was performed using a FACSCanto II (BD Biosciences, San Jose, California, USA). Data were analysed in Diva software (BD Biosciences, San Jose, California, USA). Viable lymphocytes were gated based on forward and side scatter plots of propidium-iodide staining. Subpopulations where gated using strategy as outlined in Supplementary Table S1 and Figs S1-S2, a modified version of strategies described in detail in^37–39^. Absolute cell counts were calculated in patients by combining flow cytometric proportions and lymphocyte count by a haematology automated analyser. Total lymphocyte count was not performed in controls.

### Somatic hypermutation and CD4+ T-lymphocyte stimulation

As a measurement for affinity maturation of antibodies, somatic hypermutation (SHM) was measured in kappa light-chain gene transcripts of the rearranged immunoglobulin genes using a VkappaA27-specific restriction enzyme-based hot-spot mutation assay (IgkappaREHMA) as previously described^40^. The proliferative capacity of CD4 T-lymphocytes was assessed *in vitro* by stimulating mononuclear cells labelled with carboxyfluorescein succinimidyl ester (CFSE) with anti-CD3/CD2/CD28 coated beads at 37 °C for 4 days. Subsequently, the cells were stained with fluorochrome-conjugated anti-CD4-antibodies and the CD4+ T-lymphocyte proliferation was measured by flow cytometry as dilution of CFSE during cell division. The percentage of proliferating CD4+ T-lymphocytes was recorded as the percentage of the mean proliferation in samples from two healthy controls run simultaneously with the patient sample. In our laboratory, results above 85% are considered within normal range.

### Immunoglobulins and mannose-binding lectin

Plasma concentrations of immunoglobulin (Ig) classes (M, A and G) and IgG subclasses were analyzed at Statens Serum Institute, Copenhagen, Denmark, using standardized assays. Mannose-binding lectin (MBL) serum concentrations were determined as described previously^41^.

### Clinical data

Clinical data, including date of onset of symptoms, date of admission, treatment protocol, treatment outcome, comorbidities, medical treatment one month prior to admission, results of sedimentation rate (SR), C-reactive protein (CRP), haemoglobin, white blood cell count, lymphocyte count, neutrophil count and monocyte count at diagnosis as well as results from 16s-PCR and culture of blood or biopsy samples and serologic test for Human Immunodeficiency Virus (HIV), were collected from patient records. Date of diagnosis was defined as date of MRI confirming diagnosis.

### Statistics

As data remained skewed after transformation, non-parametric statistics were performed throughout. The Wilcoxon matched-pair signed-rank test and Mann–Whitney *U* test were used, as appropriate. Unless otherwise stated, data are reported in median (IQR). Analyses were performed with the statistical software package R, version 3.3.2.

## Electronic supplementary material

Supplementary material

## Data Availability

The datasets generated during and/or analysed during the current study are not publicly available due to restrictions from the Danish Data Protection Agency but are available in anonymized form from the corresponding author on reasonable request.
