# Supplementary material for: Quantitative B-lymphocyte deficiency and increased TCRγδ T-lymphocytes in acute infectious spondylodiscitis
Source: Sci Rep. 2018 Oct 11;8:15174. doi: 10.1038/s41598-018-33318-w (PMC6181974; doi:10.1038/s41598-018-33318-w)

**Manuscript title:** Quantitative B-lymphocyte deficiency and increased TCRγδ T-lymphocytes in acute infectious spondylodiscitis

**Authors:** Anna K Haugaard^1^*; Hanne V Marquart^2^ ; Lilian Kolte^3^, Lars Peter Ryder^2^; Michala Kehrer^4^ , Maria Krogstrup^5^; Ulrik B Dragsted^6^; Benny Dahl^7,8^; Ida E Gjørup^5^; Åse B Andersen^1^; Peter Garred^2^; Susanne D Nielsen^1^

**Supplementary material**

| **Supplementary Table S1.** B and T-lymphocyte subsets, gating strategy. Viable lymphocytes were gated based on forward and side scatter plots of propidium-iodide staining. Subpopulations where gated using strategy as outlined below, a modified version of strategies described in detail in manuscript references [35-37]. | | |
| --- | --- | --- |
| **Name** | | **Marker** |
| B cell subsets | | CD19+ |
| Naïve | | CD19+ CD27- IgD+ |
| Transitional | | CD19+ CD38+++ IgM++ |
| Plasmablasts | | CD19+ CD38+++ IgM- |
| Isotype switched | | CD19+ IgM- IgD- |
| Non-isotype switched | | (CD19+ IgM+ IgD-) or (CD19+ IgM- IgD+) or (CD19+ IgM+ IgD+) |
| Memory kappa/ lambda | | CD19+ CD38+, kappa+ or Lambda+ |
| IgM only memory | | CD19+ IgM+ IgD- |
| Marginal zone like | | CD19+ CD27+ IgD+ |
| CD21 low | | CD19+ CD21(+) CD38- |
| **T cell subsets** | CD3+ | |
| CD4 | | CD3+ CD4+ |
| CD8 | | CD3+ CD8+ |
| αβ T cells | | CD3+ TCRαβ+ |
| γδ T cells | | CD3+ TCRγδ+ |
| Memory CD4 | | CD3+ CD4+CD45RO+ |
| Memory CD8 | | CD3+ CD8+CD45RO+ |
| Recent thymic emigrants CD4 | | CD31+ of CD4+CD45RA+CD45RO- |
| Naïve CD4 | | CD3+ CD4+CD45RA+ CD197+ |
| Naïve CD8 | | CD3+ CD8+CD45RA+ CD197+ |
| Effector memory CD4 | | CD3+CD4+CD45RA-, CD197- |
| Central Memory CD4 | | CD3+CD4+CD45RA-, CD197+ |
| Effector memory CD8 | | CD3+CD8+CD45RA-, CD197- |
| Central Memory CD8 | | CD3+CD8+CD45RA-, CD197+ |
| Activated CD4 | | CD3+ CD4+ HLA-DR+ |
| Activated, exhausted CD4 | | CD3+ CD4+ HLA-DR+ CD7- |
| Activated CD8 | | CD3+ CD8+ HLA-DR+ |
| Activated, exhausted CD8 | | CD3+ CD8+ HLA-DR+ CD7- |
| Tc17 | | CD8+CD196+CD161+ |
| Th17 | | CD4+CD196+CD161+ |
| Treg | | CD4+CD127-CD25+ |

| **Supplementary Table S2**. Clinical characteristics and level of CD19 B-lymphocytes in 7 cases with selective immunoglobulin deficit at follow up | | | | | | | | | | | |
| --- | --- | --- | --- | --- | --- | --- | --- | --- | --- | --- | --- |
| ID | IgM | IgA | IgG | IgG1 | IgG2 | IgG3 | IgG4 | Gender | Age | Identified microbe | CD19 B cells at follow up |
| 15 | Normal | Normal | Normal | Normal | **Low** | Normal | **Low** | Male | 34 | Atypical Mycobacteriae | Normal |
| 16 | Normal | Normal | Normal | Normal | Normal | Normal | **Low** | Woman | 51 | TB^a^ | Normal |
| 17 | **Low** | Normal | Normal | Normal | Normal | Normal | Normal | Male | 29 | *S.* *Aureus* | Normal |
| 18 | Normal | Normal | Normal | Normal | Normal | **Low** | Normal | Woman | 79 | Unknown | Normal |
| 19 | Normal | Normal | Normal | Normal | **Low** | **Low** | Normal | Male | 77 | *E.* *Coli* | Normal |
| 31 | Normal | Normal | Normal | Normal | Normal | **Low** | Normal | Male | 66 | *Streptococcus* species, *E. Coli* and *E. faecalis* | Normal |
| 34 | Normal | Normal | Normal | Normal | Normal | **Low** | **Low** | Male | 63 | Unknown | Normal |
| Normal = within reference range at follow up. Low= below reference range at follow up. ^a^This patient never received a confirmed microbial diagnosis but was treated according to typical clinical presentation of infection with TB. TB= *Mycobacterium* *tuberculosis*, *S.Aureus*= *Staphylococcus aureus, E. Coli* = *Escherichia* *Coli*, *E. faecalis* = *Enterococcus faecalis* | | | | | | | | | | | |

| **Supplementary Table S3.** Flow panel for B and T-lymphocyte subsets including reagents, with company name and product number. | | | | | | | |
| --- | --- | --- | --- | --- | --- | --- | --- |
| FITC | PE | PerCP_Cy5.5 | PE-Cy7 | APC | APC-H7 | PacificBlue | V500 |
| IgG1  BD  342409 | IgG2a  BD simultest | IgG2a  BD  550927 | IgG1  BD  348808 | IgG1  BD  345818 | IgG1  BD  560167 | IgG1  DAKO X0987 | IgG1  BD  560787 |
| TCRab  BD 333140 | TCRgd  BD 333141 | HLA-DR  BD  339216 | CD7 BeckmanCoulter PN A46526 |  | CD4  BD  560158 | CD8  DAKO  PB 984 | CD3  BD  560770 |
|  | CD31  Serotec  MCA1738PE | CD45RO  BD  560607 | CD45RA  BD  337186 |  | CD4  BD 560158 | CD8  DAKO  PB 984 | CD3  BD  560770 |
|  |  | CCR7 CD197  PerCP_Cy5.5 Biolegend 353220 | CD45RA  BD 337186 |  | CD4  BD 560158 | CD8  DAKO  PB 984 | CD3  BD  560770 |
| IgM  BD  555782 | IgD  BD  555779 | CD21  Pe-Cy5  BD 551064 | CD38  BeckmanCoulter  A54189 | IgG  BD  550931 | CD45  BD  641399 | CD19  BioLegend 302233 | CD27  BD  561222 |
| Kappa  BD simultest  349516 | Lambda |  | CD38  BeckmanCoulter  A54189 | CD20  BD  340908 |  | CD19  BioLegend  302233 | CD45  BD  560777 |
|  | CD161  MACS  130-092-677 | CD196  BD  560467 | CD25  BD  335824 | CD127  BD  558598 | CD4  BD  560158 | CD8  Dako  PB 984 | CD3  BD  560770 |
| BD= Becton, Dickinson and Company. | | | | | | | |

**Supplementary Figure S1.** Extracts from flow panels, B-lymphocyte subsets in A) a healthy control, and B) a patient example at baseline.


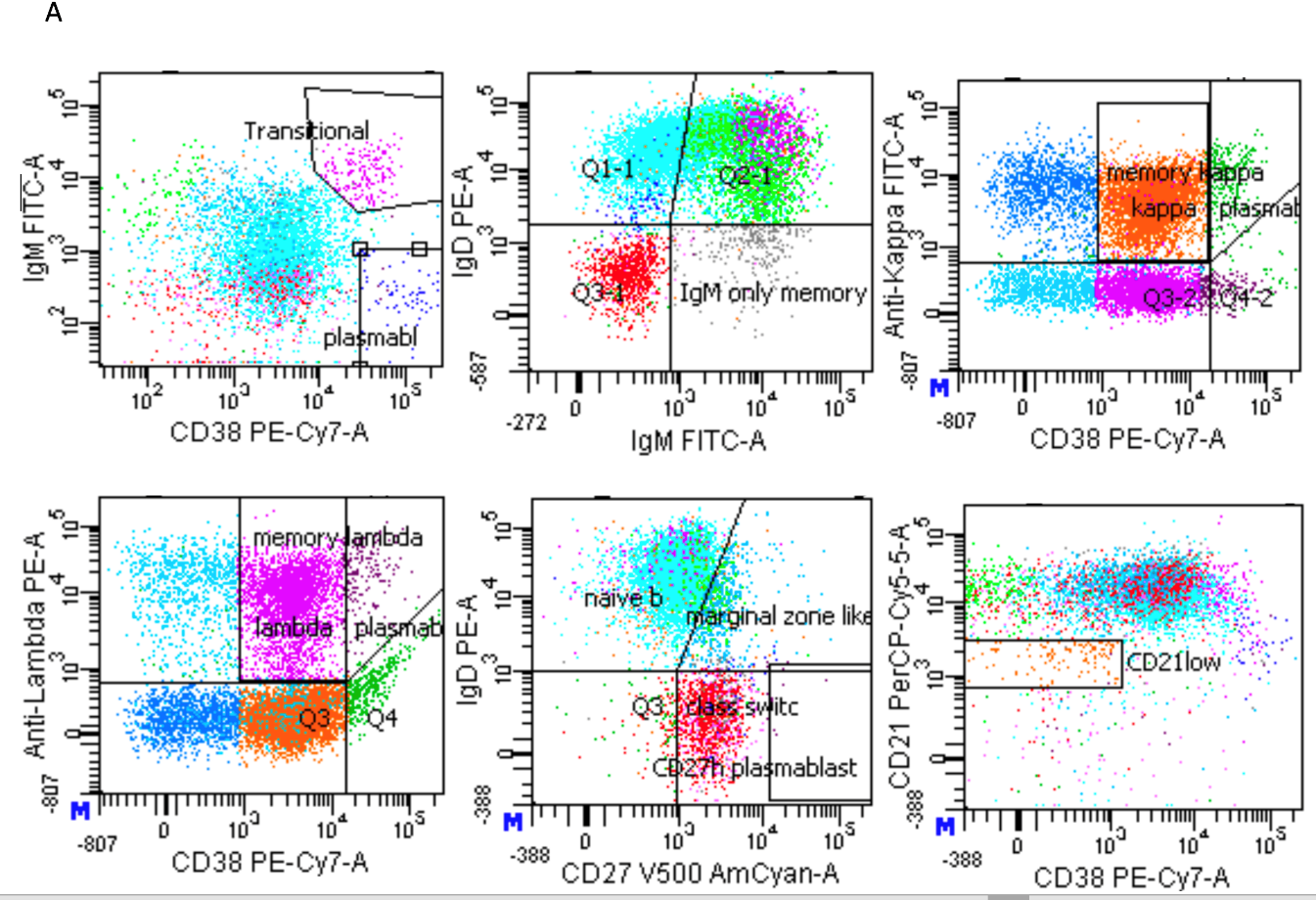

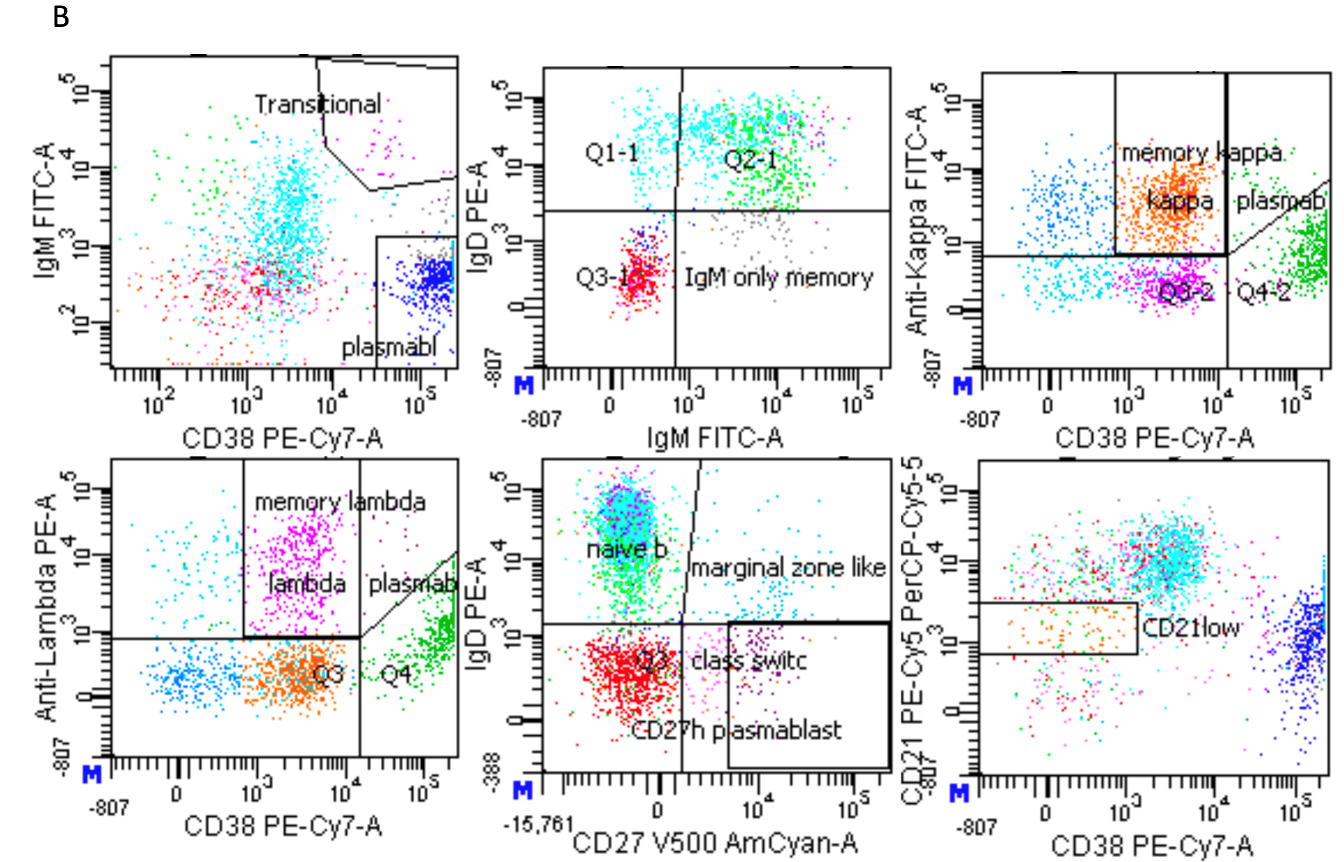


**Supplementary Figure S2.** Extracts from flow panels, T-lymphocyte subsets in A) a healthy control, and B) a patient example at baseline.


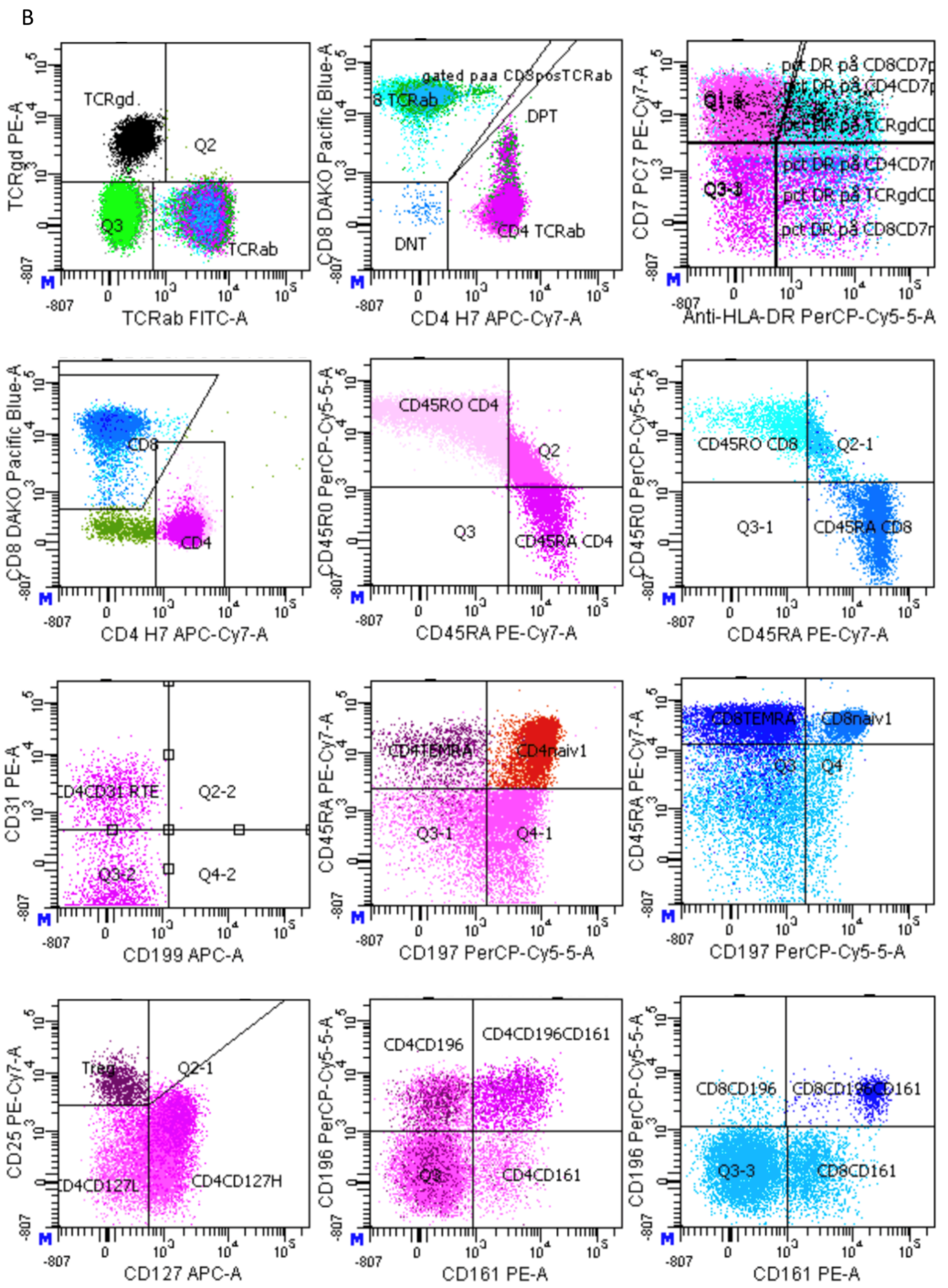

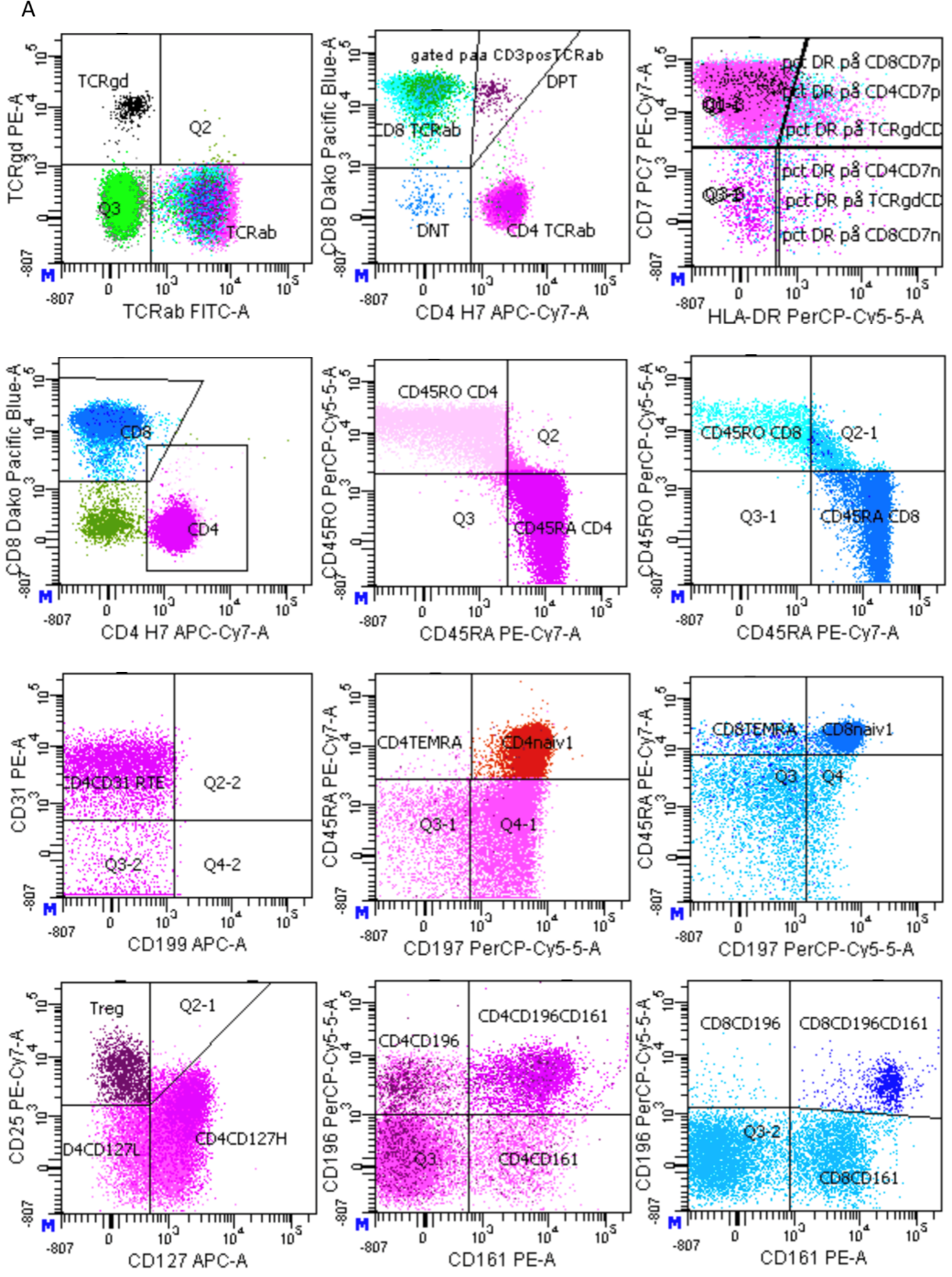

Supplement: Supplementary file 1 — Supplementary material [file 41598_2018_33318_MOESM1_ESM.docx]
